# Antibacterial Activity of Polymer Coated Cerium Oxide Nanoparticles

**DOI:** 10.1371/journal.pone.0047827

**Published:** 2012-10-26

**Authors:** Vishal Shah, Shreya Shah, Hirsh Shah, Fred J. Rispoli, Kevin T. McDonnell, Selam Workeneh, Ajay Karakoti, Amit Kumar, Sudipta Seal

**Affiliations:** 1 Department of Biology, Dowling College, Oakdake, New York, United States of America; 2 Department of Mathematics and Computer Science, Dowling College, Oakdale, New York, United States of America; 3 Department of Biology, Southern University at New Orleans, New Orleans, Louisiana, United States of America; 4 Advanced Materials Processing Analysis Center, Nanoscience Technology Center, Mechanical Materials Aerospace Eng, University of Central Florida, Orlando, Florida, United States of America; RMIT University, Australia

## Abstract

Cerium oxide nanoparticles have found numerous applications in the biomedical industry due to their strong antioxidant properties. In the current study, we report the influence of nine different physical and chemical parameters: pH, aeration and, concentrations of MgSO_4_, CaCl_2_, KCl, natural organic matter, fructose, nanoparticles and *Escherichia coli*, on the antibacterial activity of dextran coated cerium oxide nanoparticles. A least-squares quadratic regression model was developed to understand the collective influence of the tested parameters on the anti-bacterial activity and subsequently a computer-based, interactive visualization tool was developed. The visualization allows us to elucidate the effect of each of the parameters in combination with other parameters, on the antibacterial activity of nanoparticles. The results indicate that the toxicity of CeO_2_ NPs depend on the physical and chemical environment; and in a majority of the possible combinations of the nine parameters, non-lethal to the bacteria. In fact, the cerium oxide nanoparticles can decrease the anti-bacterial activity exerted by magnesium and potassium salts.

## Introduction

Cerium oxide nanoparticles (CeO_2_ NPs) are amongst the most widely used rare earth compound finding applications in industrial and commercial products. The industrial applications includes its uses as a polishing agents [1], ultraviolet absorbing compound in sunscreen [2], solid electrolytes in solid oxide fuel cells [3], as a fuel additive to promote combustion and in automotive exhaust catalyst [4]. However, it is in the biomedical industry CeO_2_ NPs is gaining much attention because of their antioxidant properties [5]. Their applications range from fighting inflammation and cancer, to radiation protection of cells [6]. Madero-Visbal et al. recently suggested that CeO_2_ NPs reduces the G-III dermatitis and skin hyperpigmentation in cells exposed to ionizing radiation [7]. Studies have also shown that CeO_2_ NPs also prevents ionizing radiation induced pneumonitis [8] and human colon cells pre-exposed to CeO_2_ NPs survived radiation insult [9]. Celardo et al. recently showed the anti-apoptotic activity of the NPs and Dowding et al. illustrated the ability of NPs to quench nitric oxide radicals [10]–[11]. In all the studies reported and reviewed in the literature, the mechanism of action is linked to the anti-oxidative property of the NPs. To expand the applications of CeO_2_ NPs, scientists are investigating methods to prevent the NP aggregation in the biological media or within the body and to improve the cellular uptake. For CeO_2_ NPs and other NPs the use of biocompatible polymers as dispersing agents is being preferred due to its low cytotoxicity [12]–[19]. Dispersing agents exhibiting cytotoxicity would prevent any biomedical applications of the NPs. Karakoti et al. have studied the antioxidant activity of PEGylated CeO_2_ NPs, whereas Perez et al. and Alili et al. have shown that dextran coated CeO_2_ NPs (dex-CNPs) have anti-tumor and anti-oxidant properties [16]–[18].

In the current study we report the antibacterial activity of dextran coated NPs (dex-CNP). Only four reports are available on the antibacterial activity of CeO_2_ NPs. Thill et al. and Pelletier et al. have reported the antimicrobial activity of CeO_2_ NPs against *Escherichia coli*, whereas Fang et al. showed the antibacterial activity against *Nitrosomonas europaea* [20]–[23]. Kuang et al. compared the toxicity of bulk CeO_2_ against the NPs counterpart and found that NP form of CeO_2_ is more toxic to *E. coli* than the bulk form [24]. In addition, the knowledge of how the toxicity or cell viability changes with the changes in the physical and chemical environment is absent. It is known that a change in the physical and chemical environment can significantly influence the toxicity of NPs [25]–[26]. Thill et al. and Pelletier et al. provide some insight on the effect of pH, growth media, and particle size and concentration of NPs on the antibacterial properties of CeO_2_ NPs [20]–[21]. These studies used non-stabilized NPs when carrying out the assay and the observed toxicity is a result of aggregated particles. In the current study, we aim at investigating the influence of nine different physical and chemical parameters (pH, aeration and, concentrations of MgSO_4_, CaCl_2_, KCl, natural organic matter, fructose, NPs and *E. coli*) on the antibacterial activity of dextran coated CeO_2_ nanoparticles (dex-CNPs).

## Results

### Characterization of NPs

**Figure 1:**
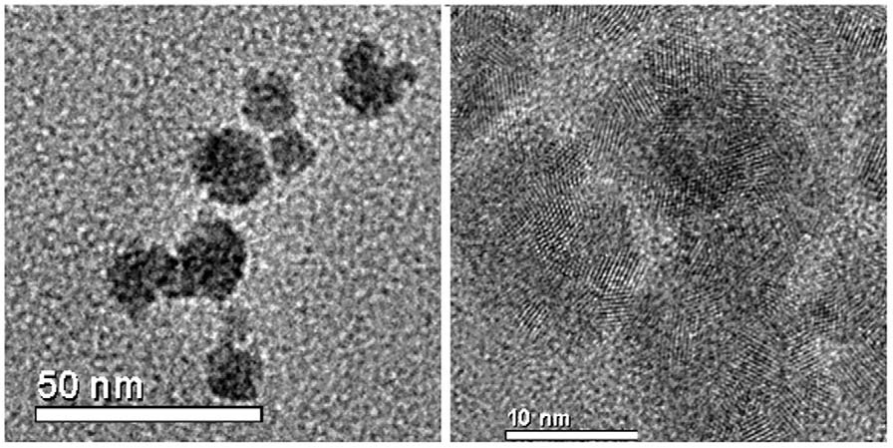
TEM images of cerium oxide complexed with dextran revealing the size of NPs to be 8–10 nm (a) High resolution TEM image showing the crystallite size of 2–4 nm (b).

**Figure 2:**
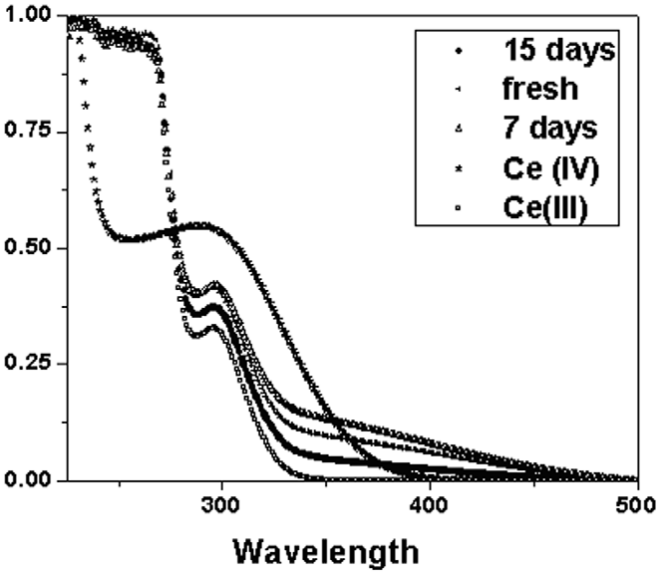
UV-visible spectrum of cerium oxide nanoparticle complexed with dextran were recorded at different time to monitor the change in valence state of cerium.

Size analysis of the CeO_2_ NPs was carried out using HR-TEM and dynamic light scattering measurement of NPs in solution. TEM images of dex-CNPs reveal the size of NPs to be 8–10 nm with the individual crystallite size of 2–4 nm (Figure 1). From the DLS measurement, the particle size in aqueous medium was 31±2.1 nm (by Vol%). The valence state of Cerium (Ce) is governed by the synthesis method of the CeO_2_ NPs, as well as the size of nanoparticle [26]. Ce can exist in a 4^+^ as well as a 3^+^ state, and the percentage of each state in an aqueous solution is dependent on the redox environment. The valence state of Ce in the dex–CNP was monitored by recording the UV-Visible spectra at regular intervals of time until it was used for the anti-bacterial activity experiments. A combination of H_2_O_2_ and nitric acid was used as the oxidizer for the Ce salt during the nanoparticle preparation, and thus the oxidation state of Ce changes from 3^+^ to 4^+^. From the revised Pourbaix diagram of Ce in the presence of H_2_O_2_/O_2_ it was noted that between a pH of 2 to 7 Ce can undergo both oxidation and reduction by the H_2_O_2_ (redox potential, E_0_ = −1.5 V). The reduction process is kinetically limited, resulting in a slow reduction of Ce as the NPs nucleate and grow from the solution. Thus we monitored the valence state of Ce in the CeO_2_ NPs with dextran in an aqueous medium. The spectrum of the fresh solution has higher Ce^+4^ oxidation state. On the subsequent measurements after 1 day, 7 days and 15 days of aging at room temperature, the Ce^+3^ state increased (Figure 2). A detailed study on surface properties of dex-CNPs has been published earlier by the author's group, where the presence of dextran on the surface of ceria NPs was demonstrated by using ATR-FTIR [27]. The surface properties of as-synthesized NPs such as surface charge and redox state of nanoceria is governed by the pH of the medium of the synthesis. Dextran is an anhydrogluco polymer of glucose and the chelating ability of polyhydroxyl groups of dextran is utilized in the synthesis of dex-CNPs, wherein multiple glucose units in the dextran complexes with cerium. Thus in the anti-bacterial activity studies, it is not expected that the coated NPs will undergo an interaction with other ions and was evaluated by using pure dextran as a control.

Cerium oxide nanoparticles synthesized in water (bare) will have different physico-chemical properties than the nanoparticles synthesized in dextran. The surface of bare nanoparticles is exposed to the ambient environment. This allows these nanoparticles to undergo changes in oxidation and chemical state in presence of various proteins and salts at a faster rate as oppose to dextran stabilized nanoparticles. Additionally, bare nanoparticles show faster agglomeration in cell culture environment as compared to the dextran stabilized nanoparticles. The accessibility of the nanoparticle surface for chemical reaction is limited by the presence of dextran coating however the small molecular weight of dextran (1000 Daltons) used herein will not behave as an impermeable barrier to the nanoparticle surface. In contrast to bare nanoparticles some of the surface cerium ions in dextran stabilized nanoparticles will be utilized in complexation with the dextran molecules and will thus be unavailable for surface reactions. We have shown previously that cerium oxide nanoparticle can complex with the polyhydroxyl compounds and influence the oxidation state of cerium ions on the surface of nanoparticles [27].

### Model Development and Validation

**Figure 3:**
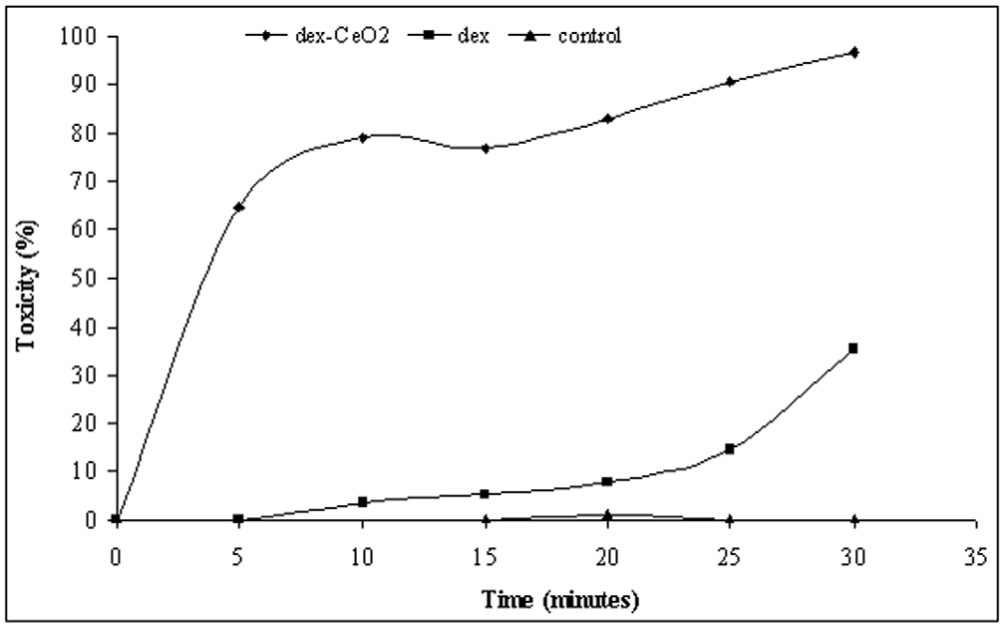
Antibacterial activity of 4.3 ppm cerium oxide nanoparticle complexed with dextran, dextran alone in comparison to control in sterile distilled water.

Figure 3 shows the antibacterial activity of 4.3 ppm dex-CNP as a function of time in sterile distilled water. More than 64% of the activity is observed within 5 minutes of incubation. Between 10 and 15 minutes of incubation, there is no increase in antibacterial activity. After 15 minutes, the increase in antibacterial activity is attributed to the activity of dextran. Thus, 15-minute time point is selected for evaluating the antibacterial activity of dex-CNP.

**Figure 4:**
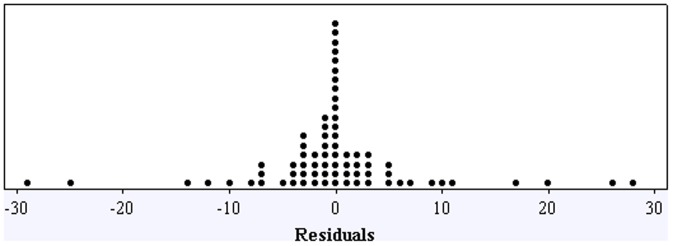
Dot plot showing the distribution of residuals for the regression model described in **Table 2**.

A statistical design of experiments involving 9 variables was designed (Table 1) and is described in detail in the method section. It is important to note that the concentration of *E. coli* is one of the tested variable (x_6_) in the statistical design. Using the anti-bacterial activity percentages shown in Table 1, a quadratic regression model (Table 2) was developed. The model has primary, secondary and interaction terms, and is a good predictor of anti-bacterial activity percentage with an R-square value of 0.913 and a standard error of 16.5. The model yields an F ratio of 3.243, which is significant with a p-value of 0.005. The distribution of residuals is symmetric and narrowly concentrated close to zero, with a standard deviation of 8.25 (Figure 4). To validate the model, five points not used in the regression analysis were selected at random (Table 3). The anti-bacterial activity values obtained in these experiments are close to the predicted values, indicating that the model has the ability to correctly predict the region of high and low anti-bacterial activity in the design space.

**Table 1:** Design of experiment for understanding the toxicity of dextran-cerium oxide nanoparticles on *E. coli* and the observed toxicity in each condition.

	x1	x2	x3	x4	x5	x6	x7	x8	x9	
	pH	[MgSO4]	[KCl]	[CaCl2]	[NOM]	[nano]	[*E. coli*]	RPM	[Fructose]	Toxicity
Variable		ppm	ppm	ppm	ppm	ppm	cells/mL	ppm	%	
Minimum	6	0	0	0	0	0	4.63E+05	0	0	
Maximum	8	15	15	15	3	4.3	4.68E+07	400	100	
**Experiment**										
1	0	0	0	0	0	0	0.12	0	0	21
2	0.5	0.5	0.5	0.5	0.5	0.5	0.61	0.5	0.5	37
3	1.5	1.5	1.5	1.5	1.5	1.5	1.60	1.5	1.5	43
4	2	2	2	2	2	2	2.10	2	2	33
5	0	2	2	2	2	2	2.10	2	2	39
6	2	0	2	2	2	2	2.10	2	2	38
7	2	2	2	0	2	2	2.10	2	2	0
8	2	2	2	2	0	2	2.10	2	2	13
9	2	2	2	2	2	2	0.10	2	2	21
10	2	2	2	2	2	2	2.10	2	0	29
11	0.5	1	1	1	1	1	1.11	1	1	13
12	1	0.5	1	1	1	1	1.11	1	1	67
13	1	1	0.5	1	1	1	1.11	1	1	0
14	1	1	1	0.5	1	1	1.11	1	1	4
15	1	1	1	1	0.5	1	1.11	1	1	24
16	1	1	1	1	1	0.5	1.11	1	1	41
17	1	1	1	1	1	1	0.61	1	1	0
18	1	1	1	1	1	1	1.11	0.5	1	99
19	1	1	1	1	1	1	1.11	1	0.5	22
20	1.5	1	1	1	1	1	0.72	1	1	24
21	1	1.5	1	1	1	1	0.72	1	1	4
22	1	1	1.5	1	1	1	0.72	1	1	0
23	1	1	1	1.5	1	1	0.72	1	1	3
24	1	1	1	1	1.5	1	0.72	1	1	0
25	1	1	1	1	1	1.5	0.72	1	1	0
26	1	1	1	1	1	1	1.02	1	1	17
27	1	1	1	1	1	1	0.72	1.5	1	0
28	1	1	1	1	1	1	0.72	1	1.5	19
29	1.5	0.5	0.5	0.5	0.5	0.5	0.39	0.5	0.5	31
30	0.5	1.5	0.5	0.5	0.5	0.5	0.39	0.5	0.5	30
31	0.5	0.5	1.5	0.5	0.5	0.5	0.39	0.5	0.5	12
32	0.5	0.5	0.5	1.5	0.5	0.5	0.39	0.5	0.5	8
33	0.5	0.5	0.5	0.5	1.5	0.5	0.39	0.5	0.5	0
34	0.5	0.5	0.5	0.5	0.5	1.5	0.39	0.5	0.5	17
35	0.5	0.5	0.5	0.5	0.5	0.5	0.94	0.5	0.5	2
36	0.5	0.5	0.5	0.5	0.5	0.5	0.39	1.5	0.5	8
37	0.5	0.5	0.5	0.5	0.5	0.5	0.39	0.5	1.5	11
38	0	2	2	2	2	0	0.11	2	2	13
39	2	0	2	2	2	0	0.11	2	2	14
40	2	2	0	2	2	0	0.11	2	2	44
41	0	0	0	2	0	0	0.11	0	0	20
42	2	2	2	2	0	0	0.11	2	2	20
43	2	2	2	2	2	0	0.11	2	2	21
44	0	0	0	0	0	0	0.83	0	0	29
45	2	2	2	2	2	0	0.11	0	2	7
46	2	2	2	2	2	0	0.11	2	0	29
47	1	1.5	1.5	1.5	1.5	1.5	0.59	1.5	1.5	7
48	1.5	1	1.5	1.5	1.5	1.5	0.59	1.5	1.5	18
49	1.5	1.5	1	1.5	1.5	1.5	0.59	1.5	1.5	18
50	1.5	1.5	1.5	1	1.5	1.5	0.59	1.5	1.5	0
51	1.5	1.5	1.5	1.5	1	1.5	0.59	1.5	1.5	0
52	1.5	1.5	1.5	1.5	1.5	1	0.59	1.5	1.5	0
53	1.5	1.5	1.5	1.5	1.5	1.5	0.65	1.5	1.5	0
54	1.5	1.5	1.5	1.5	1.5	1.5	0.59	1	1.5	0
55	1.5	1.5	1.5	1.5	1.5	1.5	0.59	1.5	1	0
56	0.5	0.5	0.5	0.5	0.5	0	0.28	0.5	0.5	19
57	1	1	1	1	1	0	0.45	1	1	22
58	1.5	1.5	1.5	1.5	1.5	0	0.62	1.5	1.5	20
59	2	2	2	2	2	0	0.79	2	2	51
60	0.5	1	1	1	1	0	0.45	1	1	7
61	1	0.5	1	1	1	0	0.45	1	1	15
62	1	1	0.5	1	1	0	0.45	1	1	0
63	1	1	1	0.5	1	0	0.45	1	1	44
64	1	1	1	1	0.5	0	0.45	1	1	25
65	1	1	1	1	1	0	0.45	1	1	31
66	1	1	1	1	1	0	0.28	1	1	13
67	1	1	1	1	1	0	0.45	1	0.5	23
68	1	1	1	1	1	0	0.45	0.5	1	30
69	0	0	2	0	2	2	1.07	2	0	27
70	0	0	2	0	2	0	1.07	2	0	29
71	0	0	0	0	2	0	1.07	2	0	24
72	2	2	0	0	1	0	0.32	0	1	64
73	2	0	0	2	0	0	0.32	0	2	62

**Table 2:** Regression coefficients of independent variables obtained by analyzing the data described in Table 1.

Parameter	Coefficient	Parameter	Coefficient	Parameter	Coefficient
x1	28.0	x2x5	−28.0	x5x9	−66.5
x2	−179.9	x2x6	−99.1	x6x7	−41.7
x3	101.1	x2x7	96.4	x6x8	9.5
x4	120.5	x2x8	−14.9	x6x9	−7.7
x5	13.2	x2x9	−18.4	x7x8	−145.9
x6	24.1	x3x4	−145.3	x7x9	11.7
x7	8.8	x3x5	57.6	x8x9	33.4
x8	−49.5	x3x6	−11.5	x1∧2	7.6
x9	−24.8	x3x7	38.0	x2∧2	113.5
x1x2	36.9	x3x8	−79.1	x3∧2	−48.0
x1x3	55.8	x3x9	69.0	x4∧2	−55.4
x1x4	6.0	x4x5	63.9	x5∧2	−11.5
x1x5	−62.5	x4x6	103.4	x6∧2	5.6
x1x6	−16.4	x4x7	−15.3	x7∧2	41.7
x1x7	14.1	x4x8	24.8	x8∧2	60.8
x1x8	−28.7	x4x9	1.6	x9∧2	18.5
x1x9	−28.0	x5x6	8.9	x10	−11.3
x2x3	31.5	x5x7	−3.5		
x2x4	−34.3	x5x8	40.5		

**Table 3:** Design of experiments used to validate the model described in Table 2 along with the predicted and obtained data.

	x1	x2	x3	x4	x5	x6	x7	x8	x9	Predicted	Observed
	pH	[MgSO4]	[KCl]	[CaCl2]	[NOM]	[nano]	[*E. coli*]	RPM	[Fructose]	Toxicity	Toxicity
Variable		ppm	ppm	ppm	ppm	ppm	cells/mL		ppm	%	%
Minimum	6	0	0	0	0	0	4.63E+05	0	0		
Maximum	8	15	15	15	3	4.3	4.68E+07	400	100		
**Experiment**											
V1	1	1	1	1	1	1	0.18	1	1	9	0
V2	1	2	1	1	1	1	0.18	1	1	61	48
V3	1	1	1	1	1	1	0.18	1	0	30	31
V4	2	2	0	0	2	0	1.6	0	2	20	21
V5	1	0	0	1	0	0	1.6	1	1	19	18

**Figure 5:**
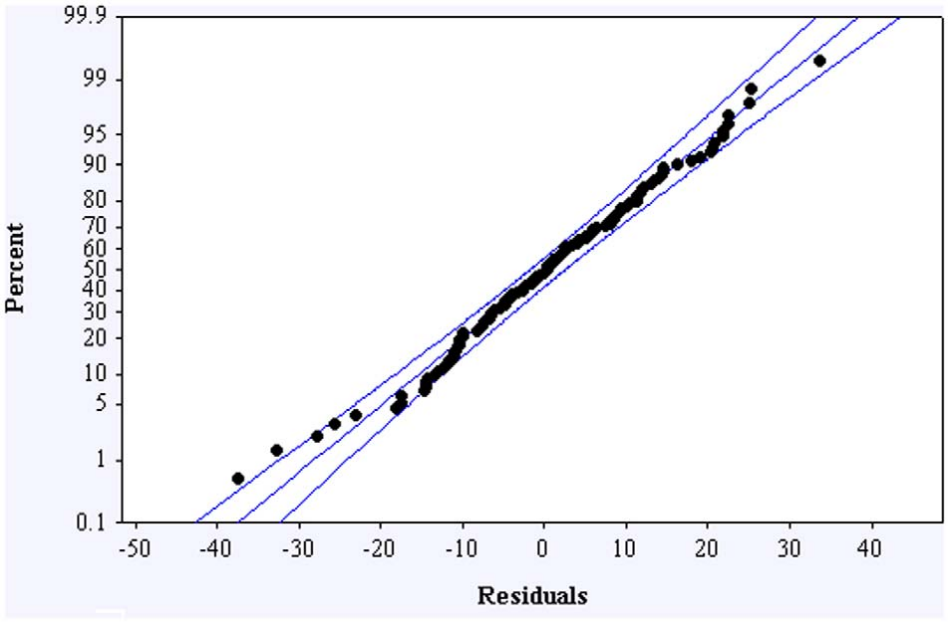
Probability plot showing the distribution of residuals along with 95% confidence range for the model described in Table S1. This model was developed by treating repeated trials as separate trials instead of averaging the results.

To confirm that the experimental differences observed in the repetition experiments do not influence the predictive reliability of the model shown in Table 2, an alternate model was obtained by treating the repeated trials as separate trials instead of averaging the results (Table S1). The probability plot shown in Figure 5 clearly shows a normal distribution of residuals confirming the low degree of variability amongst replicate data points. An analysis of the term-wise tolerances and p-values indicate that it is possible to obtain a model with fewer terms by eliminating some of the interaction terms. However, the authors are attempting to assess all of the interaction effects and are considering all possible solutions; hence all of the interaction terms have been included in the model. We did not test for three-way or higher interactions.

It is hard to elucidate the effect of each parameter considering the primary, secondary and interaction effects of each variable. However, based on model coefficients it is safe to conclude that MgCl_2_ (x_2_) is the most important input variable associated with the anti-bacterial activity of dex–CNP against *E. coli* with very high values for primary, secondary and interaction terms, especially with NPs (i.e., the x_2_x_6_ coefficient) and *E. coli* concentration (the x_2_x_7_ coefficient).

To understand the effect of each variable within the given set of conditions, the visualization is helpful (Download the Supplementary application file Application S1.zip and run it following the instructions provided in the methodology section). *E. coli* requires aeration and sugars for growth. When the concentration of cations and nanoparticles were set to 0 and 4.3 ppm respectively, changing the pH between 6 and 8, and aeration between 100 rpm and 400 rpm, shows no anti-bacterial activity related to NPs. Thus on its own, dex-CNPs are nontoxic to *E. coli*. It is important to note that CeO_2_ NPs have been known to resist broad changes in the pH [28]

**Figure 6:**
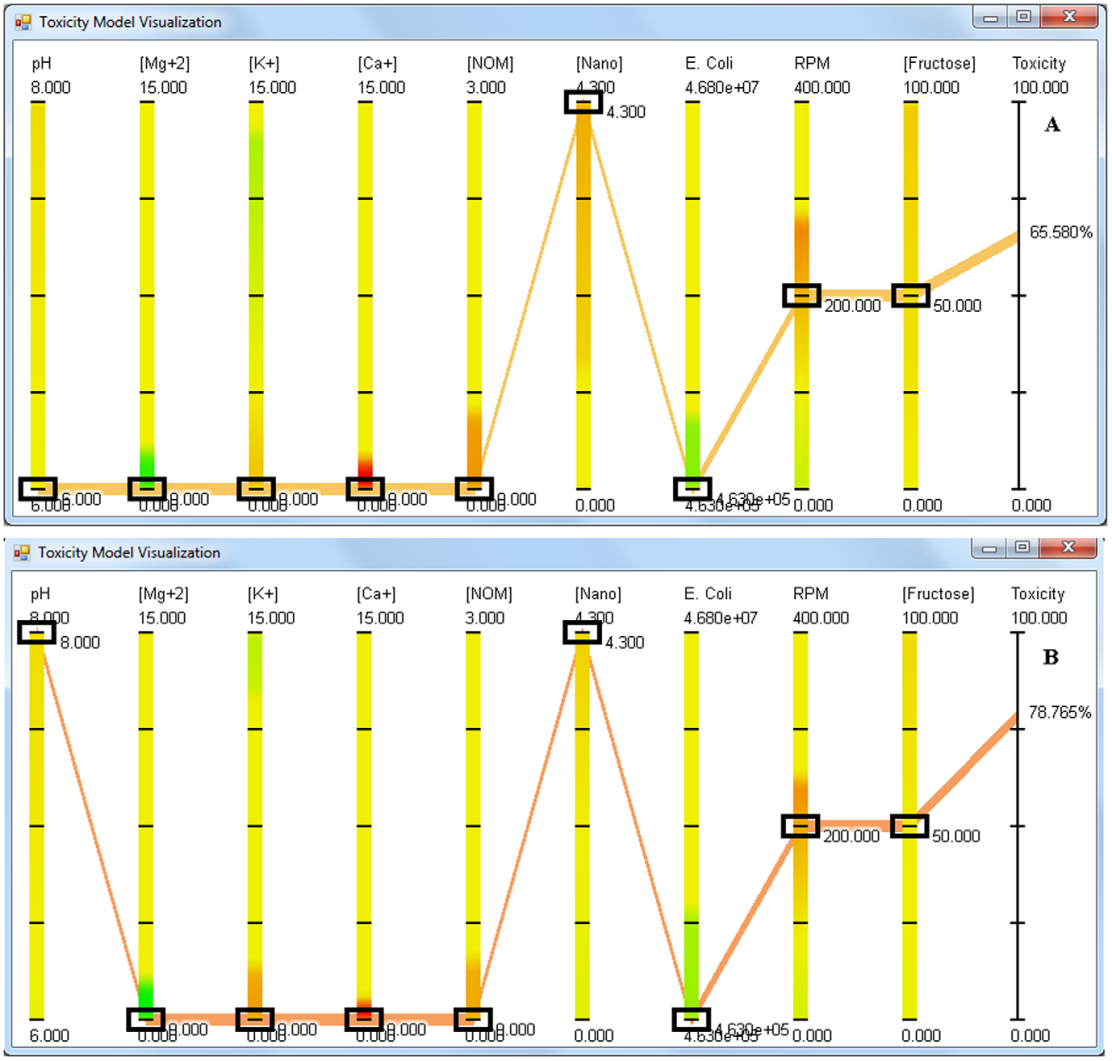
Screen shot of the visualization application showing the anti-bacterial activity of CeO_2_ NPs at a) pH 6 and b) pH 8. The concentration of cations and NOM is set to 0, NPs and *E. coli* are at 4.3 ppm and 4.63×10^5^ CFU/mL respectively whereas RPM and fructose are set at 200 RPM and 50 ppm respectively.

To understand the synergistic effect of various parameters on the behavior of dex-CNP, the first step was to set the concentrations of cations, aeration, natural organic matter (NOM) and fructose to 0 ppm, 200 rpm, 0 ppm, and 50 ppm respectively on the application. When the *E. coli* concentration is above 1×10^7^ CFU/mL, no anti-bacterial activity of dex-CNPs is observed in the pH range from 6 to 8. However, upon decreasing the concentration of *E. coli* below 1×10^7^ CFU/mL, one starts to observe the anti-bacterial activity of dex-CNPs. Figure 6a and 6b are the screen shots of the application showing anti-bacterial activity of dex-CNP under varying pH and low *E. coli* concentration. The anti-bacterial activity of 4.3 ppm dex-CNP is 65% when the pH is 6 as opposed to 0% anti-bacterial activity when no particles are present in the media. At pH 8, 75% and 72% anti-bacterial activity is observed in presence and absence of NPs, illustrating the absence of the anti-bacterial activity attributed to NPs under alkaline conditions. Thus the anti-bacterial activity of dex-CNPs is dependent on the concentration of the target cells and on the pH.

**Figure 7:**
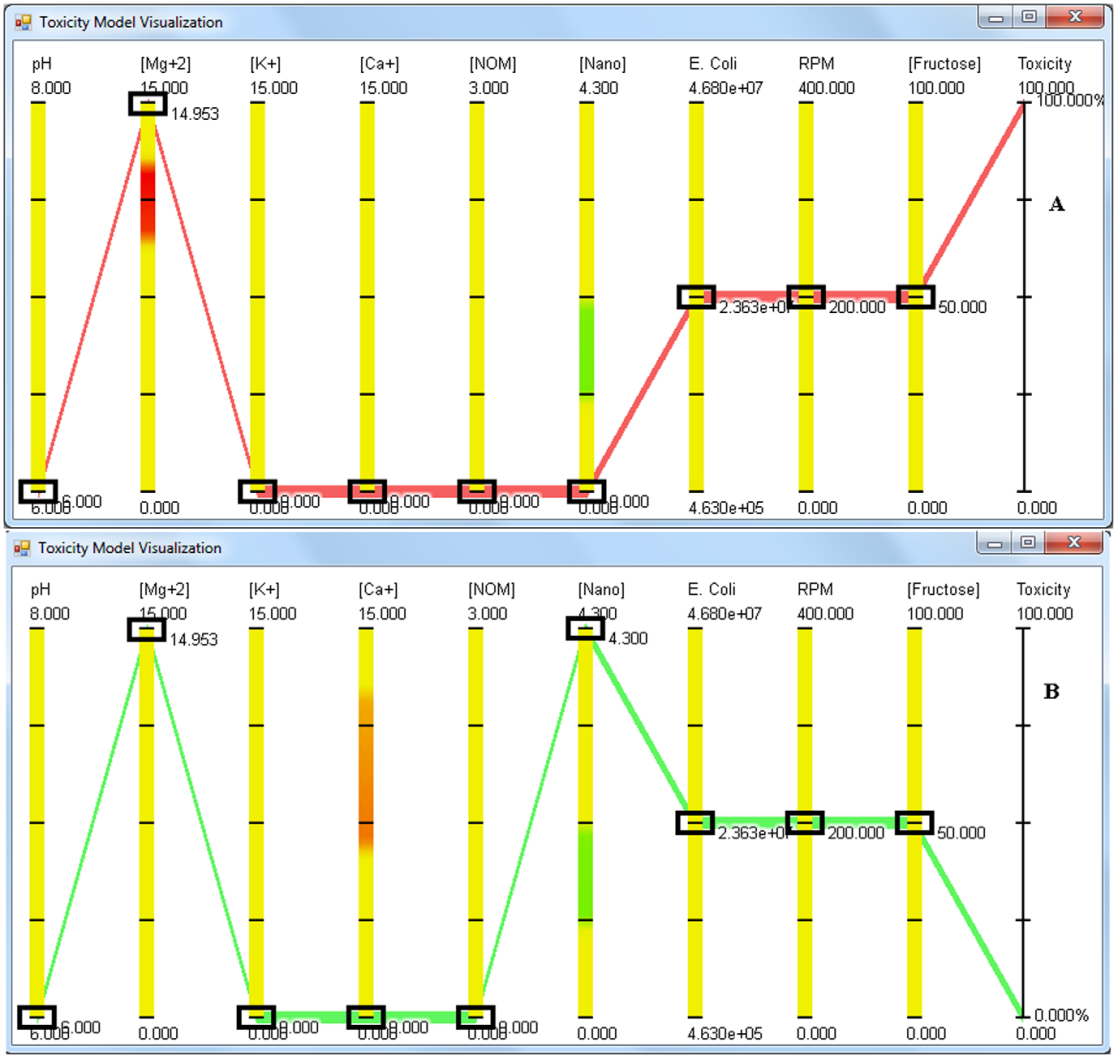
Screen shot of the visualization application showing the effect of magnesium salts on the anti-bacterial activity of CeO_2_ NPs at a) 0 ppm and b) 4.3 ppm concentration when MgSO_4_ concentration is 15 ppm. The concentrations of KCl, CaCl_2_ and NOM is set to 0, pH and *E. coli* are at 6 and 2.36×10^7^ CFU/mL respectively whereas RPM and fructose are set at 200 RPM and 50 ppm respectively.

**Figure 8:**
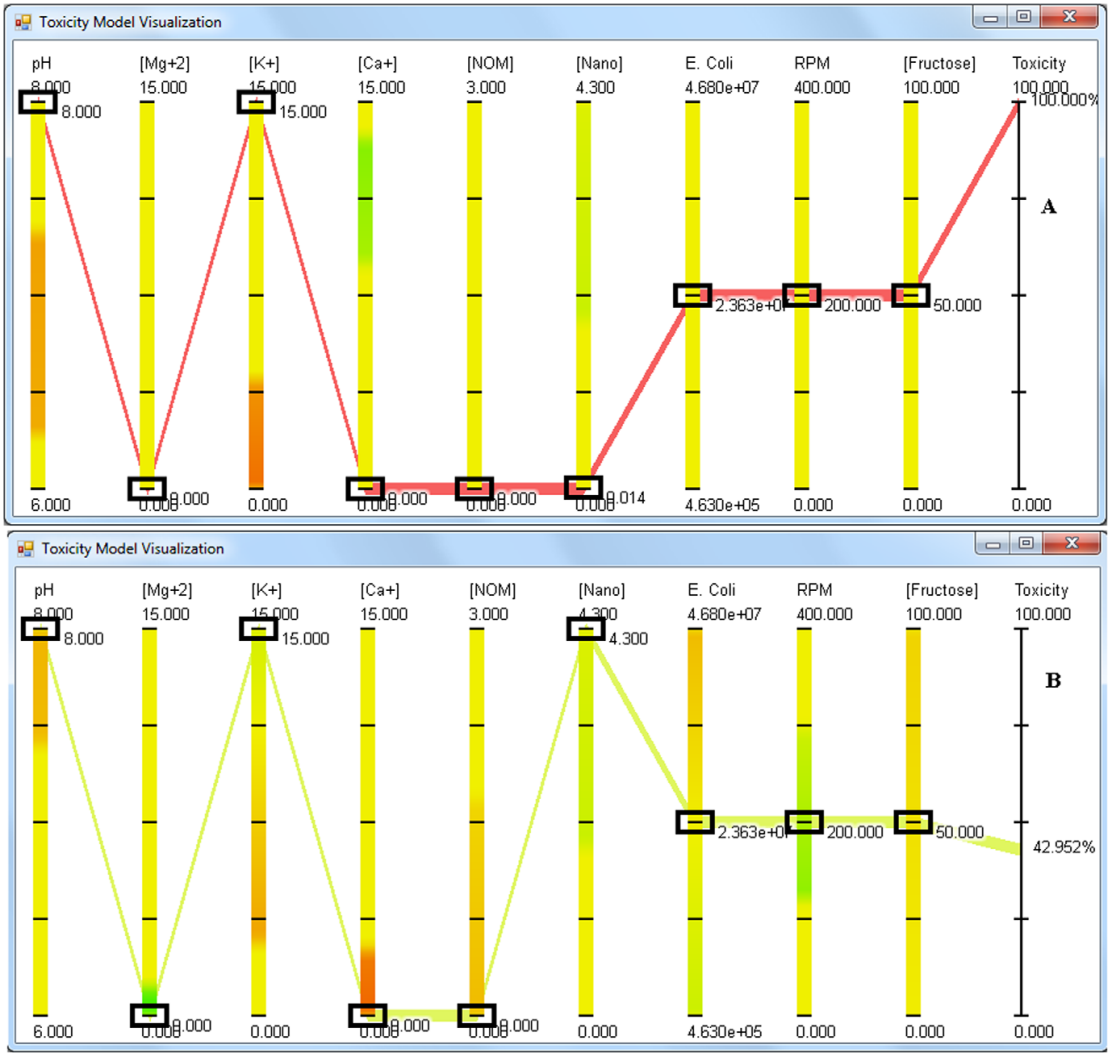
Screen shot of the visualization application showing the effect of potassium salts on the anti-bacterial activity of CeO_2_ NPs at a) pH 8, 0 ppm nanoparticle concentration, b) pH 8, 4.3 ppm nanoparticle concentration. The concentrations of MgSO_4_, CaCl_2_ and NOM is set to 0, KCl and *E. coli* are at 15 ppm and 2.34×10^7^ CFU/mL respectively whereas RPM and fructose are set at 200 RPM and 50 ppm respectively.

**Figure 9:**
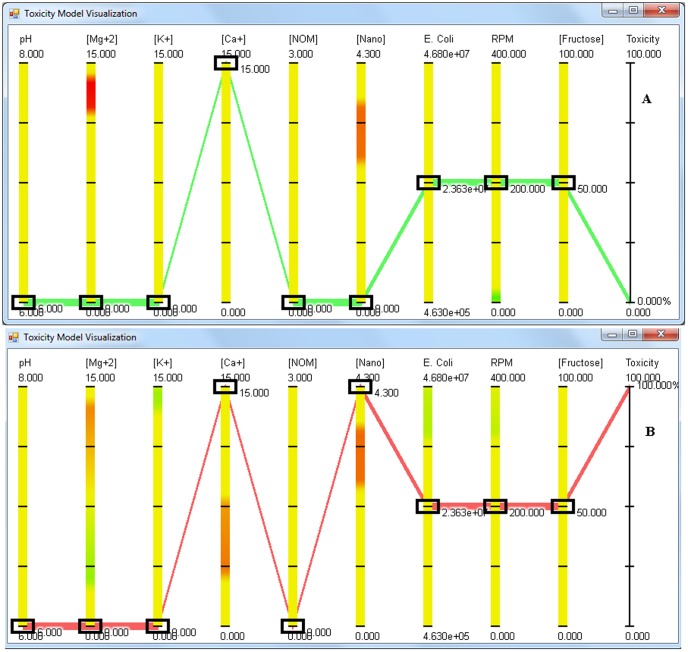
Screen shot of the visualization application showing the effect of calcium salts on the anti-bacterial activity of CeO_2_ NPs at a) 0 ppm nanoparticle concentration and b) 4.3 ppm nanoparticle concentration. The concentrations of MgSO_4_, KCl and NOM is set to 0, CaCl_2_ and *E. coli* are at 15 ppm and 2.36×10^7^ CFU/mL respectively whereas RPM and fructose are set at 200 RPM and 50 ppm respectively.

When MgSO_4_ is added to the media, after approximately 10 ppm concentration, an increase in anti-bacterial activity against *E. coli* is observed. 100% anti-bacterial activity is observed at MgSO_4_ concentration of 15 ppm, and this anti-bacterial activity is independent of pH (Figure 7a shows the screen shot of the application at pH 6). Increasing the concentration of NPs negates the negative effects of magnesium ions, with no anti-bacterial activity of MgSO_4_ observed at a dex-CNPs concentration of 4.3 ppm (Figure 7b) under the entire range of cell concentration tested. The application predicts that dex-CNPs has the ability to decrease the anti-bacterial activity of a high concentration of KCl, with 58% reduction in anti-bacterial activity observed at pH 8.0 (Figure 8a and 8b). Figure 9a shows the absence of the anti-bacterial activity of CaCl_2_ when no dex-CNPs are present and pH of 6.0. When the dex-CNPs concentration is increased to 3.5 ppm, the anti-bacterial activity reaches the maximum of 100% (Figure 9b). This trend is opposite to that observed for MgCl_2_ and KCl.

There are different reports in the literature suggesting either antagonistic or synergistic effect of NOM on the anti-bacterial activity of NPs. Suwannee river NOM, obtained from International Humic Substances Society, was used in the current study. Increasing the concentration of this NOM shows marginal increase in the anti-bacterial activity of dex-CNPs with the value of 13% when 3 ppm of NOM is added to media, along with 4.3 ppm of dex-CNP and 2.3 CFU/mL, pH 6.

The direct influence of pH and aeration on the anti-bacterial activity of dex-CNPs to *E. coli* was not observed, unless the aeration was decreased below 100 rpm and pH increased to 8.0. At this point (pH 8.0 and aeration of 0 rpm), increasing the NP concentration decreases the anti-bacterial activity. Under alkaline pH, the organism could be undergoing a change in metabolism and adapting to the new environment. It is not clear whether the observed effect of aeration is because of the increase in dissolved oxygen in the media or the effect of shaking. By increasing the NP concentration, through an unknown mechanism, the organism is better able to adapt to the alkaline pH. To elucidate the interaction between the cations, one can visualize the effect of MgCl_2_ on KCl anti-bacterial activity. The 100% anti-bacterial activity of KCl observed at pH 8 was reduced to 42% when 4.3 ppm dex-CNPs was added (Figure 9a and 5b). An addition of 3.7 ppm of Mg^+2^ completely eliminates the anti-bacterial activity.

## Discussion

The statistical design of experiments, comprising 73 experiments, was custom-designed for the current study to ensure that the design is free of any unnecessary constraints, is able to capture the interaction effects of the parameters on the anti-bacterial activity, and the total number of experiments needed is not unrealistic. Within the design, 18 design points had an x_6_ (concentration of NPs) of zero. This allows us to incorporate the control experiments within the design, thereby avoiding a separate set of experiments.

In the current study, we observed that dex-CNPs are non-toxic or exert mild anti-bacterial activity to *E. coli* under a wide range of experimental conditions tested. The CeO_2_ NPs were synthesized directly in dextran solution and were never dried from suspension, thereby keeping the oxidation state of NPs constant before anti-bacterial activity analysis. We hypothesize that the complexation of CeO_2_ NPs with dextran plays a critical role in determining the cell viability of the NPs and needs further investigation. As the NPs are coated with dextran, the direct contact between NPs and *E. coli* may not occur. This contact has been shown to be critical in determining the anti-bacterial activity of CeO_2_ NPs [20]. Further, Kunzmann et al. have recently shown that dextran-coated iron oxide NPs were not toxic to human macrophages and dendritic cells primarily because of the properties of dextran rather than that of the particles [29]. By preventing the contact with the cell, the anti-oxidative properties of the CeO_2_ NPs can be exploited for biological applications without any cytotoxicity being observed. Further studies are under way to confirm the role of surface coating on the antibacterial activity of CeO_2_ NPs and understand the reasoning behind the observed effects of physical and chemical parameters.

The ability of the dex-CNPs to decrease the anti-bacterial activity of magnesium and potassium salts can be explained by the adsorption of these ions on the particles. Such adsorption would decrease the ions that could act on the bacteria. Support for the hypothesis comes from the ability of NOM to decrease the anti-bacterial activity of MgSO_4_ similar to that of dex-CNPs. NOM is known to bind to metals and thereby decrease the eventual anti-bacterial activity of the metal ions [30]–[31]. The absence of the effect of the NOM on the antibacterial activity of the dex-CNPs could again be attributed to the presence of dextran coating, which prevents any direct interaction between the particles and the organic matter. The interaction of calcium ions with dex-CNPs is not well understood in the literature. We hypothesize that if calcium salts are added in the presence of dex-CNPs, calcium ions may decrease the binding of cerium NPs on the dextran, releasing them into the media. Once the particles are free in the media, they may come into contact with the bacteria and exert antibacterial activity.

We believe that a high degree of attention is warranted in understanding the role of biocompatible dispersing agents like dextran in the observed biological activity of NPs coated with such agents. These studies could lead us to increase biomedical applications of NPs. The current study also enforces that any bioactivity determination of a nanoparticle preparation should involve investigation under different combinations of chemical and physical parameters, as opposed to a single-parameter study. Single-parameter studies could be misleading and should be avoided. Finally, the use of interactive graphics allows us to predict the activity of NPs, or any other agents, under any combination of parameters used to develop the model. Such interactive applications could be developed for smartphones, computers, environmental sensors or other electronic appliances to allow scientists to predict the activity of a molecule in the given matrix immediately upon entering the parameter values.

## Materials and Methods

### Synthesis of NPs

The CeO_2_ NPs coated via complexation of a cerium salt with dextran were prepared by a modified procedure of aqueous preparation as described previously [26]. Briefly, a 5 mM solution of dextran (M.W 1000 amu – purchased from Sigma Aldrich) was prepared and filtered using 0.2 micron filter (Anodisc) to remove suspended and undissolved impurities. Cerium nitrate (III) hexahydrate was then dissolved in this solution and stirred for two hours to allow complete complexation to take place between the dextran and cerium ions. Stoichiometric amounts of hydrogen peroxide (30% w/w) was added to the solution as it changed color from colorless to light yellow to dark orange. The pH of the solution was maintained below 3.5 using dilute nitric acid, if necessary, to stabilize the NPs from aggregating. The final concentration of CeO_2_ NPs was 5 mM.Low molecular weight dextran was selected as the coating for the particle because of its biocompatibility. Our preliminary experiments showed that under the concentration used, it does not inhibit the growth of *E. coli*.

### UV-Visible spectrophotometry

The UV-visible spectrum data was obtained using Perkin Elmer Lambda 750 UV-VIS NIR instrument with a diffuse reflectance detector. All samples were characterized against water as the reference sample. The UV-visible measurements were recorded from fresh, seven-day aged samples and 15-days aged samples to monitor the progress of reversible changes in oxidation state of CeO_2_ NPs. UV-visible spectra were also recorded for cerium nitrate solution and ammonium cerium nitrate solution as reference for Ce(III) and Ce(IV) oxidation state.

### High-resolution transmission electron microscopy

High resolution transmission electron micrographs (HRTEM) were obtained using a FEI Tecnai F 30 microscope operated at 300 KV with a point-to-point resolution of 0.2 nm. The samples were prepared by placing a drop of dex–CNPs solution on a holey carbon coated copper grid. The TEM grids were dried overnight in a vacuum before imaging. TEM was also performed on pure dextran solutions and dextran mixed with only cerium nitrate solution (unoxidized) to eliminate any artifacts that may originate from the complexation of dextran with cerium nitrate.

### Dynamic light scattering

Particle size and zeta potential measurements were carried out using light scattering measurements from Zeta Sizer Nano (Malvern Instruments). Samples were subjected to centrifugation at 7500 rpm for 10 minutes, and the suspension was ultrasonicated for 5 minutes before measurements. No precipitation was observed during the centrifugation.

### Design of experiment

The design matrix, denoted by X, is illustrated in coded form in Table 1. The design space is a 9-dimensional hypercube with design points distributed across the design space and on the boundaries. Except for the *E. coli* concentration, all of the other eight independent variables were tested at five levels and were coded between 0 and 2 during analysis (0, 0.5, 1, 1.5 and 2). Experiments were conducted in batches to ensure that systematic artifacts and errors are not propagated. As the exact number of bacteria (x_6_) in the experiments varies from one batch to another, the values were coded upon completion of all the experiments from 0.1 to 2.1. This variable was not coded from 0 because biologically it would not be appropriate to measure anti-bacterial activity in absence of any cells. Mathematically, the design space was shifted by adding 0.1 to this variable. The shifting does not effect the least squares regression analysis other than sifting the regression curve by 0.1. The dependent variable, Y, is the observed anti-bacterial activity percentage and is an average value of two replications.

### Anti-bacterial activity studies

*E. coli* BW25113 was obtained from The Coli Genetic Stock Center at Yale University and was grown overnight at 37°C, 200 rpm in nutrient broth medium. The culture was centrifuged at 8,000 rpm (9,803 rcf) for 5 minutes washed with sterile distilled water twice to remove any bound organic and inorganic components. The washed cells were re-suspended in sterile distilled water. 1 mL of the culture suspension was added to the media prepared in 5 mM sodium phosphate buffer (pH as shown in Table 1), followed by addition of NPs to give a final concentration of chemical parameters as shown in Table 1. The flasks were incubated at the desired rpm for 15 minutes at 37°C. Serial dilutions and plating on nutrient agar were performed to determine the colony forming units (CFU) present in the media post incubation. The percent anti-bacterial activity was calculated by comparing the number of CFU present in the media after the incubation, as compared to the number of CFU at time zero.

### Developing the model

A least squares quadratic regression model (equation 1) was constructed using the coded design described in Table 1 to predict the anti-bacterial activity of NPs against *E*. coli.
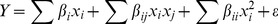
(1)Where, *Y* is the predicted response given by the anti-bacterial activity percentage. The variables *x*_1_, *x*_2_, …, *x*_9_ represent the nine input concentrations mentioned above. To improve model accuracy when predicting the change in microbial population, even in absence of NPs, a binary variable x_10_ was used to help capture this. For i = 1,2, …, 10, *β_i_*, and *β_ii_* represent linear effects and quadratic effects, respectively. For *i<j*, the coefficients *β_ij_*, represent the interaction effects. The symbol *ε* is used to denote the prediction error. This gives a total of 55 predictor terms in our model.

The regression model was obtained by solving the least-squares optimization problem: determine the set of 55 coefficients *β* = {*β_i_*, *β_ii_*, *β_ij_*} such that 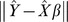 is minimum. Here 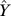 is the 73×1 vector of experimental results, 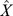 is a 73×55 augmented design matrix consisting of the original design matrix plus additional columns representing the additional predictor variables. The problem was solved using Solver, optimization software produced by Frontline and built into Excel 2007. The model coefficients obtained are given in Table 2.

It should be noted that due to the large number of variables an optimal solution to the least squares problem is not unique. In fact, depending on the starting solution for *β*, the optimal solutions obtained by Solver may vary. However, we assume that the conditions necessary among the input set to produce a particular anti-bacterial activity level are not unique as well. Hence, we view our model as one that can predict anti-bacterial activity based on the inputs, but understand that in general, there are many different condition combinations that may produce the same anti-bacterial activity percentage. Moreover, we use extreme caution when making inferences based on the coefficients obtained for our model. The final model coefficients included in Table 2, were obtained by using a starting vector of all 1's for the initial *β*. After repeating the minimization computation with Solver numerous times we observed slight variations in the optimal solutions obtained. Inferences discussed in the Results section take all of this into account.

### Interactive visual analysis

Quadratic models are widely used by the scientific community to predict the anti-bacterial activity of environmental conditions or optimize a biotechnological process [32]–[34]. However, it is not currently possible to visualize the model beyond a 3-D graph. In the current study we propose the use of an interactive graph (hereafter called “application”) for the visualization of the model. The advantages of the application includes the removal of a limitation of the number of variables that can be plotted and the elimination of the mathematical calculations involved at the user end to find the predicted anti-bacterial activity under different combinations.

The visualization application was developed using the parallel coordinates (PC) technique [35]. The application software was developed under Microsoft Windows and was written in C#, and DirectX was used for rendering. Given a regression formula that predicts a product for a given set of input parameters, the application enables the scientist to vary the experimental parameters in real-time and predict the resulting output. In our particular implementation, the dimensions of a data-set are depicted as a set of parallel vertical line segments. The lower and upper bounds on each axis are chosen by the software to correspond with the ranges of values present in the data-set for each dimension. Each experiment is visualized on these axes as a *polyline* (a set of connected line segments) that intersects each axis at the appropriate position. Overlaid on each vertical axis of the application is a color gradient that indicates how a change in that parameter affects the output. Red color indicates a positive correlation with anti-bacterial activity and green a negative one. Each small rectangular box indicates the currently selected value and serves as an interactive slider that the user can manipulate to change the parameter value. As a parameter value is increased and the slider moves into a red or orange region, the predicted anti-bacterial activity increases. Likewise, as a parameter is increased and the slider moves into a green region, anti-bacterial activity decreases. A yellowish region on an axis indicates that changes in the corresponding parameter in that region have little effect on anti-bacterial activity. A control box is also integrated in the application where one can directly input the parameter values, without the use of sliders, to obtain the predicted anti-bacterial activity values.

It is important to note that our visualization framework is very general and can be applied to other types of regression formulas with different numbers of variables and outputs and with different mathematical models. We hope that our work will encourage others who mathematically model natural systems and processes to consider using visual analysis tools to better understand complex multi-way interactions of model inputs and outputs. Systems such as ours can help the user to explore quickly the extremely large parameter space and find values of independent variables which minimize or maximize the values of dependent variable(s). Moreover, visual analysis can aid not only in prediction, which is the focus of the present work, but also in experimental design. It can suggest combinations of inputs to try experimentally to further refine the mathematical model and help the researcher to better understand the natural system itself.

### Installation Instructions for Toxicity Visualization Software

The visualization software requires that certain Windows packages be installed before the software can be run. Please follow the following steps before attempting to run the software:

1. Navigate your browser to the Microsoft Download Center to download and install Microsoft .NET Framework 3.5 Service Pack 1. This file will install components required to run certain window-driven applications on your computer. After installation, you may be required to reboot your computer.
2. Next download and install the DirectX End-User Runtime Web Installer, which will install files required to enable 3D graphics rendering on your computer. After installation, you may be required to reboot your computer.

Your computer should now be ready to run the visualization software. If the computer pops up a window asking whether to allow the program to run, you should click “Allow” to give it your permission.

## Supporting Information

Table S1 **Quadratic regression model developed obtained when the antibacterial activity observed in replicate experiments were taken as separate data point.**
(XLSX)

Application S1 **Visualization application developed using the model described in** **Table 2** **.**
(ZIP)
